# Home-based parent training for school-aged children with attention-deficit/hyperactivity disorder and behavior problems with remaining impairing disruptive behaviors after routine treatment: a randomized controlled trial

**DOI:** 10.1007/s00787-019-01375-9

**Published:** 2019-07-22

**Authors:** Ellen Nobel, Pieter J. Hoekstra, J. Agnes Brunnekreef, Dieneke E. H. Messink-de Vries, Barbara Fischer, Paul M. G. Emmelkamp, Barbara J. van den Hoofdakker

**Affiliations:** 1 Department of Child and Adolescent Psychiatry, University Medical Center Groningen, University of Groningen, Hanzeplein 1 XA10, NL-9713 GZ Groningen, The Netherlands; 2 Department of Youth Mental Health, GGZ In de Bres, Drachten, The Netherlands; 3 Jonx, Department of Youth Mental Health and Autism, Lentis Psychiatric Institute, Groningen, The Netherlands; 4 Department of Clinical Psychology, Netherlands Institute for Advanced Study, University of Amsterdam, Amsterdam, The Netherlands; 5 Department of Clinical Psychology and Experimental Psychopathology, University of Groningen, Groningen, The Netherlands

**Keywords:** ADHD, Home-based treatment, Behavioral parent training, Randomized controlled trial

## Abstract

The objective is to investigate the effectiveness of home-based behavioral parent training for school-aged children with attention-deficit/hyperactivity disorder (ADHD) and behavior problems with remaining impairing disruptive behaviors after routinely offered treatments in clinical practice. In a randomized controlled study including 73 referred children with ADHD and impairing disruptive symptoms after routine clinical pharmacotherapy and/or clinic-based parent training had been tried or, at least, offered, home-based behavioral parent training (*n* = 26*)* was compared to a waiting list (*n* = 23) and a care-as-usual home-based treatment (*n* = 24). It was unknown to families which of the home-based treatments that they received. Using mixed models for repeated measures, we examined the effectiveness on the primary outcome measure of children’s severity of disruptive behaviors and on a number of secondary outcome measures [the degree to which parents experienced the disruptive behaviors as troublesome, ADHD symptoms, oppositional-defiant disorder (ODD) symptoms, and internalizing problems]. Compared to the waiting list, children receiving home-based parent training improved significantly more regarding severity of disruptive behaviors (ES = 0.75), ADHD symptoms (ES = 0.89), ODD symptoms (ES = 0.65), and internalizing problems (ES = 0.60). Compared to care-as-usual, home-based parent training was more effective in reducing disruptive behaviors (ES = 0.57), ADHD symptoms (ES = 0.89), and ODD symptoms (ES = 0.88). Significantly more reduction of children’s internalizing problems was not found. In conclusion, children with ADHD and residual behavioral problems after routine treatment may benefit from home-based behavioral parent training.

**Electronic supplementary material:**

The online version of this article (10.1007/s00787-019-01375-9) contains supplementary material, which is available to authorized users.

## Introduction

Children with attention-deficit/hyperactivity disorder (ADHD) frequently have co-occurring disruptive behaviors [1–3] that typically form the most impairing symptoms for parents [4] and, therefore, are an important focus of treatment. Guidelines for the management of ADHD indicate medication and behavioral parent training as first choice evidence-based treatments (e.g., [5–7]). While these interventions may effectively target children’s ADHD symptoms and associated disruptive behaviors [8–10], a sizable proportion of children and families do not benefit from these treatments in clinical practice, due to a variety of reasons. Besides insufficient treatment response, this may also be due to problems of parents getting a clinic-based behavioral parent training organized, which may form a barrier to enroll in parent training programs or lead to high drop-out rates [11–13]. Other factors may also play a role in less successful behavioral parent training, such as the capacity of parents to apply the learned techniques in daily life.

The question arises what treatment should be offered to children with ADHD who still have impairing disruptive symptoms after clinic-based behavioral parent training has been offered. For this purpose, we developed a home-based behavioral parent training, based on existing evidence-based behavioral parent training programs for children with disruptive behaviors (i.e., Barkley’s Defiant Children [14], helping the Noncompliant Child of Forehand & McMahon [15], and Eyberg and Funderburk’s Parent–Child Intervention Therapy [16]) for our specific target group, delivery mode, and setting. By offering treatment at home, we aimed to increase the attendance. Cunningham and colleagues already reported higher attendance rates when parent training was located in neighborhood schools than in local clinics [17]. In addition, a home-based behavioral parent-training program for preschool children with ADHD had higher attendance than a clinic-based group behavioral parent training [18].

Besides better attendance rates, training parents in the home setting may have other advantages. A meta-analysis of Kaminski and colleagues showed that letting parents practice with their own child during treatment sessions is associated with better outcome of parent training [19]. This in vivo practice can be done more easily and more frequently when sessions take place at home. In addition, research on barriers to parent training programs has shown that parents highly appreciate flexible and individually tailored programs (see for review [12]). The home setting is particularly suitable to provide such a flexible and individually tailored approach, which may also facilitate the ability of parents to apply the behavioral techniques to their child in difficult to handle daily life situations.

The home-based treatment included not only regular behavior management techniques, but combined these with relationship enhancing strategies (following principles of Parent–Child Interaction Therapy [16]). Recent meta-analyses showed the superior effectiveness of parenting programs for reducing disruptive child behavior that integrate relationship enhancement with behavior management, compared to behavior management alone [20]. Kaminski and colleagues also showed that teaching parents to positively interact with their child is associated with improvement in parenting skills and the child’s externalizing behavior [19].

The overall aim of the present study was to investigate the effectiveness of home-based behavioral parent training for school-aged children with ADHD and behavior problems who have impairing disruptive symptoms after routine clinical pharmacotherapy and/or clinic-based parent training have been tried or, at least, offered. In a three-arm randomized controlled design, we compared the effectiveness of our home-based parent training with two different control conditions: a waiting list condition and a non-specific, care-as-usual home-based treatment. Comparison with the waiting list condition was used to investigate the effectiveness of our home-based behavioral parent training, with the expectation that this treatment would be more effective than the waiting list condition. We examined the effectiveness on children’s severity of disruptive behaviors, ADHD symptoms, oppositional-defiant disorder (ODD) symptoms, and internalizing problems and on the degree to which parents experienced the disruptive behaviors as troublesome. As these outcome measures could suffer from parent-expectation bias, we included a comparison of our home-based behavioral parent training with a non-manualized home-based treatment and also did not inform parents about the nature of the allocated home-based treatment. We expected to find effectiveness over and above non-specific and parent-expectation bias effects of the non-manualized home-based treatment. An additional aim was to investigate the long-term effects of the home-based parent training by comparing this treatment with the non-specific, care-as-usual home-based treatment at a 6-month follow-up, with the expectation to also find long-term effectiveness. We set up our study as a pragmatic effectiveness trial, i.e., fully embedded in routine clinical practice and with broad inclusion criteria, including children with and without medication, families who were not able to start or complete clinic-based parent training, as well as families who finished parent training but of whom the child did not improve sufficiently.

## Methods

### Participants and procedure

This was a three-arm, parallel, multicenter randomized controlled superiority trial with balanced randomization (1:1:1). All participants had been referred to three large child mental health care organizations in the North of The Netherlands (Accare, Jonx and GGZ In de Bres), together including 16 outpatient locations. The largest group had been referred to Accare (*n* = 60, 82.2%); just a small portion of the sample came from other sites (Jonx *n* = 4, 5.5%; GGZ In de Bres *n* = 9, 12.3%). Children had to meet the following inclusion criteria: (1) at time of referral a diagnosis of ADHD (all comorbid disorders allowed) as obtained from medical records (based on clinical interviews with the parents and teacher); (2) a Global Assessment of Functioning score of < 55, according to the DSM-IV-TR [21]; (3) current Eyberg Child Behavior Inventory (ECBI) ratings in the clinical range (i.e., intensity scale > 131 and problem scale > 3) [22]; (4) a full scale, verbal, and performance IQ > 70 as established within the previous 2 years (in 94.5% of the cases based on Wechsler Intelligence Scale for Children-III-NL); (5) had previously been offered and/or received routine treatments including ADHD medication and/or clinic-based behavioral parent training; and (6) attending primary school and aged 6–13 at time of inclusion in the trial. Children were excluded from the study if (1) they had a medical condition that prohibited participation in the study; (2) their parents were unable to understand or follow instructions, e.g., due to intellectual disability of the parents; or (3) their family had received home-based treatment in the previous year [23–25].

**Fig. 1:**
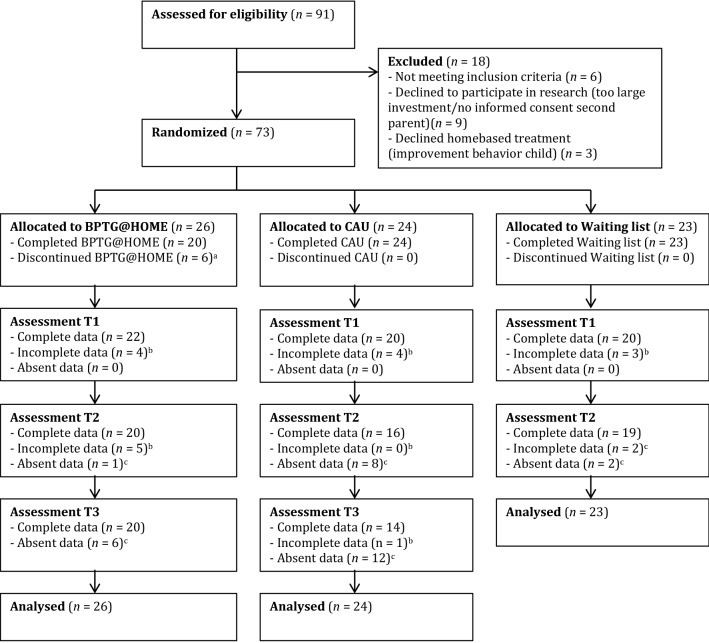
Subject flow. *n* number of children, *BPTG@HOME* behavioral parent training Groningen at home, *CAU* care-as-usual home-based parent training,* T1* baseline assessment,* T2* assessment directly after treatment or waiting list,* T3* follow-up assessment six months after both treatments. ^a^(1) parents thought treatment was no longer necessary after 1 or 2 sessions (n=4), (2) parents had other expectations of treatment (n=1), and (3) parents found treatment too intensive (n=1). ^b^ (1) parents did not return questionnaires and (2) too many missing data. ^c^Parents decline to participate in assessment

Participants who had given consent, after being informed by a member of the research project, were randomly assigned to the manualized home-based treatment (*n* = 26), a care-as-usual home-based treatment (*n* = 24), or a waiting list of 4 months (*n* = 23). Any ongoing treatment, including pharmacotherapy, was allowed across all study arms. An assistant who was not involved in the study conducted the randomization, using a computerized random number generator, and subsequently informed a member of the research team about the outcome of the randomization. Subsequently, families were provided with a sealed envelope containing a letter stating either the randomization outcome active home treatment (but not which treatment) or waiting list. Parents who were randomized to our home-based parent training or to the care-as-usual treatment were not explicitly informed about the nature of the allocated home-based treatment. Furthermore, no information about the differences between the treatments was publicized on a website and the therapists who performed the care-as-usual treatment were not informed about the content of the other home-based treatment. Moreover, when parents had questions about the randomization and the other treatment, all therapists were instructed not to answer the question, but to refer to the research team. The flow of subjects from initial recruitment through the final analysis is presented in Fig. 1. Table 1 contains child and family characteristics.

**Table 1:** Child and family characteristics (*N* = 73)

**Child characteristics**			
Age child [mean in years (SD) range]	8.8	(1.5)	6.01–12.05
Total IQ child [mean in years (SD) range]^a^	94.5	(12.3)	71–199
Male child [no (%)]	52	(71.2)	
Caucasian [no (%)]	68	(93.2)	
ADHD diagnoses assessed by clinician at time of referral [no (%)]			
ADHD combined type	48	(65.8)	
ADHD inattentive type	4	(5.5)	
ADHD hyperactive/impulsive type	11	(15.1)	
ADHD not otherwise specified	10	(13.7)	
**Family characteristics**			
Family composition [no (%)]			
Two biological parents	34	(46.6)	
One biological, one stepparent	11	(15.0)	
Single parent	28	(38.4)	
Education level mothers [no (%)]^b^			
Low	27	(37.0)	
Middle	38	(52.1)	
High	8	(11.0)	
Unknown	0	(0.0)	
Education level fathers [no (%)]^b^			
Low	18	(37.5)	
Middle	24	(50.0)	
High	5	(10.4)	
Unknown	1	(2.1)	
Siblings with identified psychopathology [no (%)]^c^	21	(28.8)	
**Treatment history**			
Pharmacotherapy for ADHD [no (%)]	68	(93.2)	
Received parent training* [no (%)]	61	(83.5)	
Finished parent training [no (%)]			
Finished training	36	(49.3)	
Drop-outs	30	(41.1)	
Unknown	7	(9.6)	
Years between parent training and inclusion in trial (*n* = 50); [mean (SD) range]	1.8	(1.6)	0.04–6.54
Other psychological treatment	38	(52.1)	
**Child characteristics and received treatment during trial**			
Comorbidity–externalizing problems [no (%)]^d^			
ODD	34	(46.6)	
CD	6	(8.2)	
Comorbidity–internalizing problems			
CBCL internalizing T-score [mean (SD) range]	63.5	(8.4)	39–82
CBCL internalizing normal range [no (%)]	19	(26.0)	
Threshold range [no (%)]	12	(16.4)	
Clinical range [no (%)]	40	(54.8)	
Current medication [no (%)]	63	(86.3)	
Change in medication during trial [no (%)]	20	(27.3)	
Other current psychosocial care at T1 [mean frequency of sessions per month (SD) range]	2.9	(4.7)	0–24
Care focused on parents	1.9	(4.3)	0–24
Care focused on child	0.9	(1.9)	0–8

*SD* standard deviation, *No* number of cases, *ADHD* attention-deficit/hyperactivity disorder, *ODD* oppositional defiant disorder, *CD* conduct disorder, *CBCL* Child Behavior Checklist

^a^Full scale IQ

^b^Classification according to a Dutch education classification system [23] which is related to the International Standard Classification of Education [24]

^c^Established by asking parents if other siblings were diagnosed with psychopathology

^d^Established with Parent Interview of Child Symptoms [25]

*All remaining parents had only attempted to start behavioral parent training

The Medical Ethical Committee of the University Medical Center in Groningen provided ethical approval for the study (METC nr 2010.289). The trial has been registered at https://www.trialregister.nl: Home-based behavioral treatment for ADHD; NTR3021. Because of the slow recruitment, we changed from a single-center to a multi-center studies.

### Measures

The primary outcome measure of our study was the ECBI, a parent-report measure of child disruptive behaviors, such as noncompliant, aggressive, hyperactive, and impulsive behaviors as well as attention problems. The Intensity scale assesses the frequency of 36 behavioral problems at home in the past week on a scale ranging from never (1) to always (7). The Problem scale measures whether the parents experienced these disruptive behaviors as troublesome in the past week (0 = no; 1 = yes). The ECBI has generally good psychometric properties (supported by more than 20 studies across cultures and countries [26, 27]. We used the ECBI as primary outcome measure, because it appears to be sensitive in measuring treatment effects [28, 29] and describes a broad range of disruptive behaviors (not only ODD and conduct disorder symptoms, but ADHD symptoms as well) which are the main focus of the treatment.

Secondary outcome measures included: (1) severity of ADHD symptoms (18 items) and (2) severity of ODD symptoms (8 items), both rated by parents with the Swanson, Nolan, and Pelham Questionnaire (SNAP-IV) on a four-point scale, ranging from not at all to very much [30]. We used the SNAP-IV to measure specific ADHD symptoms and ODD symptoms separately. As the SNAP-IV is a frequently used measure in ADHD studies (e.g., [31–34]), comparison with other trials and samples may be possible. (3) Internalizing problems were assessed with the Internalizing subscale of the Child Behavior Checklist (CBCL) [35]. Prior randomized controlled trials have found significant effects of clinical-based behavioral parent training on parent-rated internalizing problems of the child [36, 37].

All outcome measures were administered before randomization (T1; baseline) and directly after the treatment or waiting list period (T2). At T1, we also assessed ODD and CD comorbidity with the Parent Interview for Child Symptoms (P.I.C.S.-4) [25]. The time between T1 and T2 for the waiting list and our home-based treatment groups was around 4 months. The time between T1 and T2 for the care-as-usual treatment group was much longer and had greater variation, as the treatment duration was flexible. The primary outcome measure (ECBI) was also administered 6 months after both active treatment conditions (T3), but not in the waiting list group. Due to ethical restrictions we could not withhold home-based treatment from families allocated to the waiting list for longer than 4 months (after the waiting period, families received one of the two home-based treatments). All assessments took place at home of the families. A week before the assessment date, parents received a letter with the questionnaires. In the assessment visit, we checked if the questionnaires were completed and if not, we asked parents to fill them in during the visit. The primary caretaker filled in the questionnaires; in most cases (97.3%), this was the mother.

### Treatments, therapists and treatment integrity

We developed a manualized, cognitive-behavioral home-based treatment for children with ADHD and behavior problems (called “behavioral parent training Groningen at home [BPTG@HOME]”). The treatment consisted of 14–16 weekly home visits (90–120 min per session), spread over 4 months. Between every home visit, the therapist and parents had two contacts through telephone or email in which homework assignments were evaluated. In most home sessions, parents received feedback using video recordings on the skill they had been practicing that week. Furthermore, psychoeducation and cognitive interventions were given.

The treatment was divided into different treatment modules. The first module was an assessment module in which the therapist determined which treatment modules and how many sessions per module should be given. This was based on treatment goals of the family, rating scales, and the therapist’s assessment during the first session. The subsequent three basic modules: Following, Leading, and Compliance were provided to all families. These modules included three-to-six sessions, the number of sessions depending on whether the therapist considered the parents needed more practice of a certain skill. The module Following aimed to improve the parent–child relationship through daily playtime with the child. In this module, parents were taught to establish a positive interaction with their child through praise, being friendly and attentive, and avoiding instructions, questions, and criticism. Eight families (31%) received the minimum number of three sessions, for 15 (57%), 2 (8%), and 1 (4%) families and this was extended with one, two, and three sessions, respectively. In the module Leading, parents learned to prevent disruptive behavior of their child. Twelve families (46%) received the minimum number of three sessions; for 11 (42%) and 3 (12%) families, this was extended with one and two sessions, respectively. The last basic module, Compliance, taught parents how to cope with noncompliant behavior of their child. Eleven families (42%) received the minimum number of three sessions; for 11 (42%) and 4 (16%) families, this was extended with one and two sessions, respectively. After the basic modules, the therapist could select additional treatment modules, which focused on remaining parenting problems, such as disagreement between parents regarding how to handle their child or how to deal with their child’s internalizing problems (see Table 2). Twelve families (46%) received one additional treatment module and four families (15%) received two modules. After a maximum of 15 sessions, the treatment was finished with a final session in which the therapy was evaluated and in which parents planned future goals. Within 3 months, two follow-up sessions at home, aimed at maintenance of learned skills, were planned. Table 2 shows the outline of the treatment, and in the web appendix, a more detailed overview of the treatment is presented.

**Table 2:** Outline of the content of BPTG@HOME

Treatment module	Obligatory sessions	Additional sessions
**Basic modules**		
Assessment 2 sessions	1. Assessment 2. Treatment plan	
Following 3–6 sessions	1. Play time 2. Parental cognitions 3. Practicing play time	
		4. Practicing play time extended 5. Practicing play time extended 6. Practicing play time extended
Leading 3–6 sessions	1a. Providing structure	
		1b. Providing structure extended
	2. Praise 3a. Communication skills	
		3b. Communication skills extended 4. Providing structure/Praise/ Communication skills extended
Compliance 3–5 sessions	1. Assessment of noncompliant behavior 2a. House rules	
		2b. House rules extended
	3a. Reward and punishment	
		3b. Reward and punishment extended
**Additional modules**		
Disagreement between parents regarding handling the child 0–2 sessions		1. Dealing with conflicts 2. Communication with spouse
Aggressive parental behaviors to the child 0–2 sessions		1. Dealing with parental frustration 2. Dealing with parental frustration extended
Anxious or depressed child 0–2 sessions		1. Dealing with an anxious child 2. Dealing with a child with depressive symptoms
Other problem behaviors of the child 0–*x* sessions		1a. Dealing with specific problem behavior 1 1b. Dealing with specific problem behavior 1 extended 2a. Dealing with specific problem behavior 2 2b. Dealing with specific problem behavior 2 extended
**Final basic module**		
Maintenance training 3 sessions	1. Evaluation 2. Follow-up 1 3. Follow-up 2	

The basic modules were based on the principles of Barkley’s Defiant Children [14], the program of Forehand & McMahon (Helping the Noncompliant Child [15]), and Parent–Child Interaction Therapy [16]. The optional modules included basic behavioral interventions (such as exposure for child anxiety and behavior activation for depressive symptoms) and couples’ therapy techniques. Seven families (27%) received the additional module Aggressive parental behaviors towards the child, five families (19%) received the module Other problem behaviors of the child, three families (12%) received the module Anxious or depressed child, and one family (4%) received the module Disagreement between parents regarding handling the child.

Psychologists who provided BPTG@HOME were experienced in regular behavioral parent training (years of experience in regular parent training *M* = 3.5, SD = 1.5, range 2–6). However, they had no experience with home-based parent training. They all had received a 2-day training and weekly supervision from an experienced cognitive-behavioral therapist. During the study, the therapists and study staff met weekly to monitor treatment integrity; problems with adherence to the treatment manual were discussed and resolved. Furthermore, therapists completed a treatment integrity checklist after each session. Parts of the protocol that had not been covered were scheduled for the next session. Adherence to the protocol was high: the content covered in the treatment sessions was 94.3%. Non-adherence was mainly a result of the clinician’s judgment that a small part of a treatment session required adjustment or was not applicable to the case at hand.

Families in the care-as-usual condition received an uncontrolled home-based treatment, which was eclectic and had no restrictions in duration, number of sessions, or content. In The Netherlands, this care-as-usual treatment is a regular treatment for families with children with various behavior problems. However, the effectiveness of this treatment has not yet been investigated in a randomized controlled trial. The treatment started with a 4–6-week observation at home, aimed at building a therapeutic relationship with the family, assessing the problems of child, parents, and siblings, and determining treatment goals. This was followed by a non-manualized treatment phase, in which different techniques and interventions could be used, such as psycho-education, techniques from solution-focused and family therapy, and skills training (e.g., training of parenting skills or children’s social skills). Therapists were free to choose techniques and interventions. In general, the therapist visited the family once to twice every week, for 1.5–2 h. Continuation or discontinuation of the treatment was decided jointly by parents and therapist every 3 months. The care-as-usual treatment was performed by experienced social workers (years of experience with home-based treatment: *M* = 4.4 SD = 2.0, range 1.5–8.0), who had weekly peer supervision meetings.

During the 4-month waiting period, the families did not receive any form of home-based treatment.

### Sample size

We originally aimed to include *n* = 40 families in each treatment arm. With this sample size, we would have been able to detect between-group differences with an effect size (Cohen’s *d*) of 0.4 with 90% power. Unfortunately, the recruitment was much more problematic than we had thought, due to lower referral to the study than expected, resulting in *N* = 73 included patients after 3 years (March 2011–May 2014) of recruiting. Still, with this sample size the power was above 80% as recalculated with GLIMMPSE [38] for most outcome measures (ECBI Intensity scale 0.52, ECBI Problem scale 0.90, secondary outcome measures 0.81–0.99).

### Statistical analysis

#### Handling of missing data and outliers

When participants had more than 10% missing values on a questionnaire or 20% missing values on a relatively small subscale (e.g., the SNAP ODD subscale), the total scores were not calculated, but regarded as missing. In case of less missing values, the missing values were replaced by the average rating-per-item (sub)scale score, with which total scores were calculated. Using Cook’s distance, we assessed potential outliers on all outcome measures.

#### Baseline differences between study arms

We presented the change of extra care during trial (medication and other care) for each study arm. With one-way analyses of variance (ANOVA; *p* < 0.05) for continuous variables and Chi-square tests (*p* < 0.05) for categorical variables, we determined if there were significant differences between study arms with regard to basic demographic features (children’s age and IQ, and educational level of the primary caregiver), baselines scores on outcome measures, characteristics of treatment (duration of treatment, number of sessions), medication change during trial, and extra care received during the trial.

#### Effectiveness analyses

Outcomes of the treatments were analyzed with linear mixed effects models for repeated measures (MMRM), following the intent-to-treat paradigm. We investigated the short-term effectiveness for all outcome measures by comparing the mean changes from baseline to T2 of the home-based behavioral parent training with those of the waiting list and of the care-as-usual treatment. Long-term effectiveness was explored by the mean change on the ECBI from baseline to T3 of BPTG@HOME compared with that of the care-as-usual treatment, comparing ECBI ratings in the BPTG@HOME group with the care-as-usual home-based group at T3. In the MMRM analyses, the variables treatment, observation time, and the interaction between observation time and treatment (time*treatment) were entered as fixed effects, with the intercept specified as a random effect, using an unstructured covariance matrix for within-patient correlation (*p* < 0.05). The effectiveness of the treatment was assessed by interpreting the time*treatment interaction effect, reflecting the short- or long-term change in outcome measure due to a specific treatment. The random intercept in turn controls for the variety present in the baseline scores on the outcome measures, ensuring that we do not confound patient heterogeneity with treatment effects. Effect sizes were computed by dividing the mean differences between BTPG@HOME and the waiting list/care-as-usual groups at T1 and T2/T3 by the pooled sample standard deviation [39].

#### Robustness checks

We performed two sensitivity analyses. First, when significant differences were found in children’s age and IQ, educational level of the primary caregiver, the duration of the treatment, the number of sessions, frequency of medication changes during the trial, and/or amount of extra care received between study arms, additional MMRM analyses were performed by re-estimating the model with these variables as additional fixed effects. According to Peduzzi and colleagues [40], a maximum of two variables can be included given the small sample size. When more than two variables showed a significance difference between study arms, we prioritized differences in treatment characteristics (duration of treatment and number of session). When necessary, the other significant variables were included in additional analyses combined with one of the two treatment characteristics. Second, we repeated the MMRM analyses using the subsample of *N* = 61 children who previously received parent training.

## Results

### Outliers

No evidence of outliers was found, as Cook’s distances varied between 0.00 and 0.09 (ECBI intensity scale), 0.00 and 0.08 (ECBI problem scale), 0.00 and 0.06 (SNAP ADHD scale), 0.00 and 0.10 (SNAP ODD scale), and 0.00 and 0.15 (CBCL internalizing scale). This is well below the threshold of 1 [41].

### Baseline differences between study arms

Table 3 shows basic demographic features (child’s age, total IQ, and educational level of the primary caregiver), treatment characteristics (duration of treatment and number of sessions), and change of extra care during trial (medication and other care) of the three study arms. There were no significant differences between BPTG@HOME and the other study arms on the baseline ratings of the outcome measures. In addition, there were no differences in medication use during treatment between the three study arms and in extra care received at T1, T2, and T3. However, the care-as-usual treatment lasted significantly longer than BPTG@HOME and consisted of significantly more sessions, and the BPTG@HOME condition had younger children (see Table 3).

**Table 3:** Participant and treatment characteristics for each study arm

	BPTG@HOME	Care-as-usual	Waiting list
Age of child in years (*M* (SD) [range])	8.2 (1.5) [6.0–11.1]	9.0 (1.3) [6.0–11.1]	9.4 (1.4) [7.0–12.1]*
Full scale IQ of child (*M* (SD) [range])	93.4 (11.1) [71–119]	84.8 (14.0) [73–118]	95.5 (12.0) [72–199]
Educational level mother (Frequency of those with low level, middle level, and high level)	11, 13, 2	8, 14, 2	8, 11, 4
Duration of treatment in months (M (SD) [range])	5.3 (1.7) [1.3–8.8]	13.9 (5.4) [5.7–28.8]*	4.3 (.4) [3.8–5.5]*
Total number of sessions (*M* (SD) [range])	13.1 (5.1) [1–17]	29.8 (18.0) [1–87]*	
Changes in medication use during treatment (Frequency of lowered dosage, increased dosage, and change of type of medication)	5, 2, 4	2, 2, 1	1, 3, 0
Other psychosocial care at pre-treatment (mean frequency of sessions per month (SD) [range])	3.6 (4.7) [0–16.5]	2.5 (4.4) [0–16]	2.4 (5.1) [0–24]
Care focused on parents	2.4 (3.9) [0–13]	1.8 (4.0) [0–16]	1.6 (5.1) [0–24]
Care focused on child	1.2 (2.1) [0–8]	0.8 (2.0) [0–8]	0.8 (1.4) [0–4]
Other psychosocial care at post-treatment (mean frequency of sessions per month (SD) [range])	3.7 (4.5) [0–20]	4.8 (7.10) [0–23]	-
Care focused on parents	2.7 (4.1) [0–17]	4.2 (7.1) [0–23]	–
Care focused on child	0.9 (1.9) [0–8]	0.6 (1.5) [0–5]	–
Other psychosocial care at follow-up (mean frequency of sessions per month (SD) [range])	3.8 (3.9) [0–16]	3.0 (2.9) [0–8]	–
Care focused on parents	2.2 (3.7) [0–16]	1.2 (1.4) [0–4]	–
Care focused on child	1.6 (2.1) [0–6]	1.8 (3.0) [0–8]	–

*M* mean, *SD* standard deviation, *No* number of cases

*Significant difference (*p* < 0.05) with the BPTG@HOME group according to ANOVA, all other comparisons (according to ANOVA or chi-square test) were not significant

### Effectiveness analyses

Table 4 shows the results from the MMRM analyses of short- and long-term effectiveness of BPTG@HOME compared to the waiting list condition and the care-as-usual treatment. On the short run (T2), compared to the waiting list condition, BPTG@HOME was associated with larger reductions of the ECBI Intensity scale, the SNAP ADHD scale, the SNAP ODD scale, and the CBCL Internalizing scale. Except for the CBCL Internalizing scale, similar larger reductions on these scales were observed of the BPTG@HOME group compared to the care-as-usual treatment. Of further note, the T2 mean scores on the ECBI Intensity scale (133.3) in the BPTG@HOME group but not those in the other arms (143.0 and 141.8 in the care-as-usual treatment and waiting list, respectively), neared the clinical cut-off score (< 131), indicating that the disruptive behaviors post-BPTG@HOME treatment were almost below the clinical range. The T2 mean score of the BPTG@HOME group and the waiting list group on the ECBI Problem scale, but not those of the care-as-usual treatment arm reached the clinical cut-off score (< 15). On the long run (T3) and compared to the care-as-usual treatment, in the BPTG@HOME group, we observed a significantly larger reduction in the ECBI Problem scale. Furthermore, the T3 mean scores of the BPTG@HOME treatment group on the ECBI Intensity and the ECBI Problem scale both dropped below the clinical cut-off score.

**Table 4:** Outcome of BPTG@HOME vs. waiting list and care-as-usual treatment, analyzed with linear mixed model for repeated measures

	BPTG@HOME	Care-as-usual	Waiting list	BPTG@HOME vs. waiting list				BPTG@HOME vs. care-as-usual			
				*β*	*p*	95% CI	ES	*β*	*p*	95% CI	ES
**Baseline (*M* (SD) [range])**											
ECBI Intensity scale	156.1 (26.6) [103–209]	156.0 (22.1) [117–204]	149.7 (18.5) [110–182]								
ECBI Problem scale	19.9 (7.4) [3–30]	21.3 (5.7) [9.3–32]	18.0 (6.6) [5–34]								
SNAP ADHD scale	34.3 (9.6) [16–46]	31.4 (8.9) [16–51]	30.6 (9.6) [5–51]								
SNAP ODD scale	13.2 (5.2) [5–22]	11.0 (4.6) [3–22]	11.9 (5.2) [0–22]								
CBCL Internalizing scale	14.6 (6.6) [6–36]	15.2 (8.6) [1–39]	11.5 (7.4) [0–27]								
**After treatment (*M* (SD) [range])**											
ECBI Intensity scale	133.3 (27.1) [78–188]	143.0 (28.0) [99–192]	141.8 (22.1) [92–178]	17.8	0.048*	5.0–30.5	0.75	13.7	0.009**	0.1–27.2	0.57
ECBI Problem scale	14.2 (9.0) [0–26]	17.4 (8.4) [5–31]	14.6 (7.7) [3–34]	2.8	0.167	− 1.1–6.7	–	2.2	0.300	− 1.9–6.4	–
SNAP ADHD scale	24.5 (11.0) [6–50]	31.6 (11.6) [11–50]	28.5 (9.1) [7–50]	8.7	0.001**	3.8–13.6	0.89	9.3	0.001**	4.1–15.5	0.89
SNAP ODD scale	7.5 (4.7) [0–17]	10.1 (5.2) [2–19]	9.1 (4.5) [0–16]	3.2	0.028*	0.4–6.1	0.65	4.4	0.005**	1.4–7.4	0.88
CBCL Internalizing scale	8.7 (5.0) [1–18]	12.1 (8.3) [2–29]	9.8 (8.0) [1–36]	4.4	0.011*	1.1–7.6	0.60	2.6	0.139	− 0.9–6.1	–
**6 months follow-up (*M* (SD) [range])**											
ECBI Intensity scale	125.1 (21.4) [87–160]	133.0 (22.62) [94–170]	–	–	–	–	–	9.9	0.130	− 2.9–22.9	–
ECBI Problem scale	8.7 (7.4) [0–23]	13.8 (6.3) [5–23]	–	–	–	–	–	4.6	0.039*	0.4–8.8	0.65

*M* mean, *SD* standard deviation, *95% CI* 95% confidence interval, *ES* effect size, *ECBI* total score on the subscales of the Eyberg Child Behavior Checklist, *SNAP* total score on the subscales of the Swanson, Nolan and Pelham-IV, *CBCL* total score on the subscale of Child Behavior Checklist

*p < 0.05; **p < 0.01

### Robustness checks

The care-as-usual treatment lasted significantly longer than BPTG@HOME and consisted of significantly more sessions (see Table 3). Therefore, we repeated the MMRM analyses, now controlling for treatment duration and the number of sessions. At T2, we again found significantly larger reductions in the BPTG@HOME group on the ECBI Intensity scale (*β* = 14.8; *p* = 0.047; 95% CI 0.2–29.4), on the SNAP ADHD scale (*β* = 9.3; *p* = 0.002; 95% CI 3.9–14.6), and on the SNAP ODD scale (*β* = 4.6; *p* = 0.008; 95% CI 1.3–7.8). The significant decrease at T3 in the ECBI Problem scale also persisted (*β* = 4.8; *p* = 0.024; 95% CI 0.6–9.0). As an additional sensitivity analysis, we replaced either the covariate ‘duration of treatment’ or ‘number of sessions’ with ‘age’. All the results were unchanged under these new specifications.

The analysis with the subsample of *N* = 61 children who had previously received clinic-based parent training resulted in similar findings, except for the comparison between the BTPG@HOME and the care-as-usual treatment: the change on the SNAP ODD scale was no longer significant (*β* = 3.7, *p* = 0.061).

## Discussion

This study investigated the effectiveness of home-based behavioral parent training, as follow-up treatment for school-aged children with ADHD and behavior problems who had impairing disruptive symptoms after routine clinical pharmacotherapy and/or clinic-based behavioral parent training had been tried or offered. Our results suggest that home-based behavioral parent training is effective for these difficult-to-treat families: compared to a waiting list, the treatment significantly reduced children’s disruptive behaviors, ADHD symptoms, ODD symptoms, and internalizing problems, with moderate (disruptive behaviors, ODD symptoms, and internalizing problems) to large (ADHD symptoms) effect sizes. The severity of disruptive behaviors of the children in the home-based behavioral parent training group almost dropped below the clinical range directly after treatment. Prior RCTs that investigated the effectiveness of behavioral parent training for school-aged children with ADHD found positive results regarding the reduction of ADHD symptoms, disruptive behaviors, and internalizing problems as well (e.g., [36, 37, 42-47]). However, we are the first who showed this effect for a difficult-to-treat group by offering it as a follow-up intervention after routine treatments have been tried or, at least, offered. Noteworthy, we found higher effect sizes on ADHD symptoms (ES 0.89) and disruptive behavioral problems (ES 0.65–0.88) compared to prior meta-analyses (ADHD symptoms ES 0.35–0.68; disruptive behaviors ES 0.26–0.59) [9, 10, 48].

The home-based behavioral parent training was also more effective than our care-as-usual home-based treatment in reducing disruptive behaviors (moderate effect size), ADHD symptoms, and ODD symptoms (large effect sizes). As the evaluation of both active treatments may have been affected by parent-expectation bias (which we tried to minimize by not informing families about the nature of the allocated treatment), this finding suggests that the effectiveness of home-based behavioral treatment was not solely a result of parent-expectation bias. Between the home-based parent training group and the care-as-usual group, no differences in reductions of internalizing problems and improvements in experiencing the disruptive behaviors as troublesome by the parents were present. However, the care-as-usual treatment lasted on average twice as long as the home-based parent training (13.9 vs. 5.3 months, respectively) and involved twice as many home visits (29.8 vs. 13.1 visits).

While our study did not investigate which elements of the home-based parent training contributed specifically to its effectiveness, it is unlikely that only the treatment setting (at home) was crucial for the effectiveness of our program, given that the care-as-usual treatment was home-based as well, but less effective than the home-based parent training. It could be hypothesized that the use of live modeling, and video feedback during the home sessions were important components as well [49, 50]. In addition, the opportunity for parents to directly practice their parenting skills with their child during the session may have been a crucial component [19]. However, also other treatment elements may have played a role in the effectiveness of the home-based treatment, such as the length and number of sessions, and the fact that the program was individually tailored for each family. Furthermore, it cannot be ruled out that the differences in education level, working experience, and degree of supervision between the therapists of the two treatments have partially driven our results. Possibly, the treatment of this difficult-to-treat subgroup of children with disruptive behaviors requires higher educated staff with expert supervision.

We also found preliminary evidence for the effectiveness of home-based parent training in the long run. Six months after the end of treatment, children allocated to the home-based parent training group remained on average below the clinical range regarding the severity of disruptive behaviors. In addition, parents in this group experienced the disruptive behaviors as significantly less troublesome compared to the care-as-usual group. The latter result was not found directly after treatment. Possibly, first, the behavior of the child needed to improve, before the parents could adjust their cognitions about the behavior of their child in a more positive way.

High drop-out rates are common in behavioral parent training and limit their effectiveness [11, 51, 52]. With respect to our home-based behavioral parent training program, 26.4% of the eligible participants did not start the treatment or dropped out prematurely. This percentage is markedly lower than the 51% reported in a recent systematic review on parental engagement in behavioral parent training [11]. It is plausible that this lower drop-out can be attributed to offering the treatment at home; another home-based behavioral parent training for preschool children with ADHD also showed a relatively low drop out [18].

## Limitations

This study should be interpreted in light of certain limitations. First, our small sample size, which was lower than planned, should be acknowledged. Having three different treatment arms may have been less suitable given our modest sample size. Another consequence of the small sample size was that we did not take into account changes in medication during treatment, nor the reasons why the families did not profit from routinely offered treatments. However, even with a smaller sample size, we still had sufficient power and found significant results in favor of the home-based parent training. Second, we were unable to collect post-treatment assessments in one-third of the participants who had received the care-as-usual treatment. It is conceivable that those missings were from families who may have responded less well to the treatment. Thus, we may have somewhat underestimated the effectiveness of home-based parent training compared to care-as-usual. Third, the CAU home-based treatment had a much longer duration than our home-based behavioral parent training. However, controlling for treatment duration and the number of sessions did not alter our findings. Finally, we fully relied on parent-self reports as outcome measure. Recent meta-analyses recommended the use of raters who are blinded to treatment allocation (e.g., clinicians, teachers) to control for the potential bias of parent-reported measures [9, 10]. While blinded raters may be desirable when the objective of the study is generalizability across situations, such a measure may be too strict when the goal of the treatment is to improve behavioral problems in a specific context (e.g., at home) that falls outside the scope of the blinded raters. The current study took into account possible parent-expectation bias by including an active control group in the study design and not revealing to participating families the type of the assigned home-based treatment. This approach enabled the usage of parent-reported measures in a more reliable way. However, we acknowledge that we did not check whether we were successful in keeping parents unaware of the differences between the two home-based treatments.

## Future research

Apart from replicating our findings in a larger sample, future studies should try to identify essential program components and moderators of treatment effectiveness. In particular, assessing the added value of the different treatment modules and the extended sessions would be important. Furthermore, it would be worthwhile to investigate whether the home-based treatment is also effective when delivered by social workers rather than psychologists. Delivering the program by social workers would obviously make the treatment less expensive and easier to implement. Finally, we investigated home-based parent training as a follow-up treatment in a stepped-care ADHD treatment program. Unfortunately, we lack baseline scores at the time of initial treatment. We only systematically collected ratings after regular treatment had been offered. Future stepped-care studies may want to also collect response to initial treatment and/or determine its effectiveness as first or second line treatment.

## Electronic supplementary material

Below is the link to the electronic supplementary material.

Supplementary file1 (DOCX 30 kb)
