# Correction to: Home-based parent training for school-aged children with attention-deficit/hyperactivity disorder and behavior problems with remaining impairing disruptive behaviors after routine treatment: a randomized controlled trial

**DOI:** 10.1007/s00787-022-02102-7

**Published:** 2022-10-31

**Authors:** Ellen Nobel, Pieter J. Hoekstra, J. Agnes Brunnekreef, Dieneke E. H. Messink-de Vries, Barbara Fischer, Paul M. G. Emmelkamp, Barbara J. van den Hoofdakker

**Affiliations:** 1 Department of Child and Adolescent Psychiatry, University Medical Center Groningen, University of Groningen, Hanzeplein 1 XA10, NL-9713 GZ Groningen, The Netherlands; 2 Department of Youth Mental Health, GGZ In de Bres, Drachten, The Netherlands; 3 Jonx, Department of Youth Mental Health and Autism, Lentis Psychiatric Institute, Groningen, The Netherlands; 4 Department of Clinical Psychology, Netherlands Institute for Advanced Study, University of Amsterdam, Amsterdam, The Netherlands; 5 Department of Clinical Psychology and Experimental Psychopathology, University of Groningen, Groningen, The Netherlands

## Correction to: European Child and Adolescent Psychiatry (2020) 29:395–408 10.1007/s00787-019-01375-9

There is an error in Table 4, the SNAP ADHD value for the waiting list is given as 18.5 in the published paper. However, this should be 28.5.

**Table 4:** Outcome of BPTG@HOME versus waiting list and care-as-usual treatment, analyzed with linear mixed model for repeated measures

	BPTG@HOME	Care-as-usual	Waiting list	BPTG@HOME vs. waiting list				BPTG@HOME vs. care-as-usual			
				*β*	*p*	95% CI	ES	*β*	*p*	95% CI	ES
**Baseline (*M* (*SD*) [range])**											
ECBI Intensity scale	156.1 (26.6) [103–209]	156.0 (22.1) [117–204]	149.7 (18.5) [110–182]								
ECBI Problem scale	19.9 (7.4) [3–30]	21.3 (5.7) [9.3–32]	18.0 (6.6) [5–34]								
SNAP ADHD scale	34.3 (9.6) [16–46]	31.4 (8.9) [16–51]	30.6 (9.6) [5–51]								
SNAP ODD scale	13.2 (5.2) [5–22]	11.0 (4.6) [3–22]	11.9 (5.2) [0–22]								
CBCL Internalizing scale	14.6 (6.6) [6–36]	15.2 (8.6) [1–39]	11.5 (7.4) [0–27]								
**After treatment (*M* (*SD*) [range])**											
ECBI Intensity scale	133.3 (27.1) [78–188]	143.0 (28.0) [99–192]	141.8 (22.1) [92–178]	17.8	0.048*	5.0–30.5	0.75	13.7	0.009**	0.1–27.2	0.57
ECBI Problem scale	14.2 (9.0) [0–26]	17.4 (8.4) [5–31]	14.6 (7.7) [3–34]	2.8	0.167	− 1.1–6.7	–	2.2	0.300	− 1.9–6.4	–
SNAP ADHD scale	24.5 (11.0) [6–50]	31.6 (11.6) [11–50]	28.5 (9.1) [7–50]	8.7	0.001**	3.8–13.6	0.89	9.3	0.001**	4.1–15.5	0.89
SNAP ODD scale	7.5 (4.7) [0–17]	10.1 (5.2) [2–19]	9.1 (4.5) [0–16]	3.2	0.028*	0.4–6.1	0.65	4.4	0.005**	1.4–7.4	0.88
CBCL Internalizing scale	8.7 (5.0) [1–18]	12.1 (8.3) [2–29]	9.8 (8.0) [1–36]	4.4	0.011*	1.1–7.6	0.60	2.6	0.139	− 0.9–6.1	–
**6 months follow-up (*M* (*SD*) [range])**											
ECBI Intensity scale	125.1 (21.4) [87–160]	133.0 (22.62) [94–170]	–	–	–	–	–	9.9	0.130	− 2.9–22.9	–
ECBI Problem scale	8.7 (7.4) [0–23]	13.8 (6.3) [5–23]	–	–	–	–	–	4.6	0.039*	0.4–8.8	0.65

*M* mean, *SD* standard deviation, *95% CI* 95% confidence interval, *ES* effect size, *ECBI* total score on the subscales of the Eyberg Child Behavior Checklist, *SNAP* total score on the subscales of the Swanson, Nolan and Pelham-IV, *CBCL* total score on the subscale of Child Behavior Checklist

*p < 0.05; **p < 0.01

