# Supplementary material for: Home-based parent training for school-aged children with attention-deficit/hyperactivity disorder and behavior problems with remaining impairing disruptive behaviors after routine treatment: a randomized controlled trial
Source: Eur Child Adolesc Psychiatry. 2019 Jul 22;29(3):395–408. doi: 10.1007/s00787-019-01375-9 (PMC7056677; doi:10.1007/s00787-019-01375-9)
Supplement: Supplementary file 1 — Supplementary file1 (DOCX 30 kb) [file 787_2019_1375_MOESM1_ESM.docx]

**Appendix Behavioral Parent Training Groningen for children with behavioral problems - at home (BPTG@HOME)**

A brief overview of the manual [1].

| General description | |
| --- | --- |
| Treatment goals | - To decrease children’s externalizing behaviors;  - To decrease children’s internalizing problems;  - To increase parental knowledge of ADHD and comorbid problems;  - To enhance parenting skills and competence;  - To enhance parental problem-solving skills;  - To change parental dysfunctional beliefs about their child;  - To enhance positive communication between family members. |
| Treatment characteristics | - Home-based and consisting of 14-16 weekly home visits (90-120 minutes per session) and two telephone or email contacts after every home visit (first contact within 24 hours after each home visit);  - Manualized and including standard basic modules (consisting of two to six sessions), as well as additional modules (consisting of a maximum of two sessions) which can be used individualized: the therapist determines which additional treatment modules and how many sessions per module will be given;  - The basic modules are based on the parent training treatment manuals of Barkley [2], Forehand & McMahon [3], and Eyberg & Funderburk [4]; the additional modules consist of basic cognitive behavioral therapy methods as well as family and marital therapy techniques;  - Including the following main elements: psycho-education, stimulus control and contingency management techniques, relationship enhancing strategies, therapist guided video-feedback, homework assignments. The therapist needs to have a directive therapeutic approach. |

| Treatment structure and content | | |
| --- | --- | --- |
| Basic modules | Sessions | Content |
| Assessment  2 sessions | 1. Assessment  2. Treatment plan | 1. Establishing treatment goals and assessing family functioning based on rating scales and observation/video of play time  Between sessions: determine which treatment modules and how many sessions per module shall be given  2. Discussing treatment plan with parents |
| Following   - 1. sessions | 1. Play time  2. Parental cognitions  3. Practicing play time  4-6. Practicing play time extended^1^ | 1. Education on relationship enhancing techniques; use of therapist modeling during play time; parents practice with techniques during play time, using video feedback; homework: daily practice of learned techniques during play time  2. Identify and change dysfunctional beliefs of parents about relationship enhancing techniques; parents practice techniques during play time with video feedback; homework: daily practice of learned techniques during play time  3. Parents practice techniques during play time, using video feedback; homework: daily practice of learned techniques during play time  4-6. Parents practice techniques during play time, using video feedback; homework: daily practice of learned techniques during play time |
| Leading  3-6 sessions | 1a. Providing structure  1b. Providing structure extended^1^  2. Praise  3a. Communication skills  3b. Communications skills extended^1^  4. Providing structure/ Praise / Communication skills extended^1^ | 1a. Education on the need for a structured environment for children with ADHD and disruptive behaviors; structuring the environment in space and/or time  1b. Structuring the environment in space and/or time  2. Education on increasing the child’s self-esteem through praise; practicing reinforcing positive behaviors through praise, using video feedback  3a. Education on the need for clear communication/commands for children with ADHD and disruptive behaviors; practicing commands/preparing child for specific troublesome situations, using video feedback  3b. Practicing commands/preparing child for specific troublesome situations, using video feedback  4. Structuring the environment in space and/or time / practicing praise with video feedback / practicing commands/preparing child for specific context, using video feedback |
| Compliance   - 1. sessions | 1. Assessment of noncompliant behavior  2a. House rules  2b. House rules extended^1^  3a. Reward and punishment  3b. Reward and punishment extended^1^ | 1. Education on the relationship between ADHD and oppositional behaviors; filming oppositional behavior of the child at a challenging moment of the day (for parents)  Between sessions: therapist conducts functional analysis (Antecedents-Behavior-Consequences) of child’s and parents filmed behaviors  2a. Video feedback on previous session; education on the need for clear house rules for children with ADHD and disruptive behaviors; practicing installing (new) house rules, using video feedback  2b. Practicing installing (new) house rules, using video feedback  3a. Education on reinforcement techniques and punishment; designing token system; implementing token system, using video feedback  3b. Designing token system; implementing token system, using video feedback |
| Additional modules | Sessions | Content |
| Disagreement between parents regarding handling the child   - 1. sessions | 1. Dealing with conflicts  2. Communication with spouse | 1. Education on effects of parental conflicts on the child; assessment of parental conflicts; practicing to prevent parental conflicts  2. Filming parental discussion – psycho-education: communication rules – Practicing communication rules with video feedback |
| Aggressive parental behaviors towards the child   - 1. sessions | 1. Dealing with parental frustration  2. Dealing with parental frustration extended^1^ | 1. Education on effects of aggressive parental behaviors on the child; assessing aggressive behaviors of the parent(s); practicing prevention and dealing with frustrations  2. Practicing prevention of parental aggression and dealing with parental frustrations |
| Anxious or depressed child  0-2 sessions | 1. Dealing with an anxious child  2. Dealing with a child with depressive symptoms | 1. Education on the relationship between ADHD and anxiety; assessing anxiety of the child; practicing parental skills for helping the child with anxiety  2. Psycho-education relationship ADHD and depressive symptoms; assessing depressive symptoms of the child; practicing parental skills for helping the child with depressive symptoms |
| Other problem behaviors of the child  0-x sessions | 1a. Dealing with specific problem behavior 1  1b. Dealing with specific problem behavior 1 extended^1^  2a. Dealing with specific problem behavior 2  2b. Dealing with specific problem behavior 2 extended^1^ | 1a. Filming and assessing specific problem behavior of the child; designing intervention plan based on functional analyses of behavior; implementing intervention plan  1b. Evaluating intervention plan of specific problem behavior; adjusting intervention plan as appropriate  2a. Filming and assessing second specific problem behavior of the child; designing intervention plan based on functional analyses of behavior; implementing plan of action  2b. Evaluating plan of action of specific problematic behavior; adjusting intervention plan as appropriate  …. |
| Final basic module | Sessions | Content |
| Maintenance training  3 sessions | 1. Evaluation  2. Follow-up 1  3. Follow-up 2 | 1. Evaluation of the treatment with the parents; evaluation of the treatment with the family; anticipating future problems  2. Rehearsal and maintenance training; anticipating future problems  3. Rehearsal and maintenance training; anticipating future problems |

^1^ Extended means repeating the particular home visit with same content.

| General structure home visits |
| --- |
| Presentation of the agenda of current home visit |
| Discussion of homework assignments |
| Introduction of new topic through education |
| Practicing new skills (using therapist video feedback) |
| Identify and change possible dysfunctional beliefs of the parents |
| Preparation of new homework assignment |

**References**

1. Nobel E, Van der Veen-Mulders L (2012) BPTG@HOME: Behavioral Parent Training Groningen for children with behavioral problems – at home. Groningen: Accare University Center of Child and Adolescent Psychiatry, unpublished manual (available upon request)

2. Barkley RA (1987) Defiant children: A clinician’s manual for parent training. New York: Guilford Press.

3. Forehand RL, McMahon RJ (1981) Helping the noncompliant child: a clinician’s guide to parent training. New York: Guilford Press.

4. Eyberg SM, Funderburk BW (2011) Parent-Child Interaction Therapy protocol. Gainesville, FL: PCIT International
